# Assessing the Rates and Reasons of Elective Surgical Cancellations on the Day of Surgery: A Multicentre Study from Urban Indian Hospitals

**DOI:** 10.1007/s00268-021-06364-1

**Published:** 2021-11-16

**Authors:** Bhakti Sarang, Geetu Bhandoria, Priti Patil, Anita Gadgil, Lovenish Bains, Monty Khajanchi, Deepa Kizhakke Veetil, Rohini Dutta, Priyansh Shah, Prashant Bhandarkar, Lileswar Kaman, Dhruva Ghosh, Kavita Mandrelle, Ashwani Kumar, Akshay Bahadur, Sunil Krishna, Kamal Kishore Gautam, Ya Dev, Manisha Aggarwal, Neil Thivalapill, Nobhojit Roy, On Behalf of the IndSurg Collaboration, Kavita Mandrelle, Ashwani Kumar, Sunil Krishna, Lovenish Bains, Sameer Kadam, Lileswar Kaman, Dnyanesh Belekar, Anita Gadgil, Akshay Bahadur, Kamal Kishore Gautam, Parvez David Haque, Ritu Jain, Sahir Bhatti, Alisha Bhatt, Dhruva Ghosh, Manisha Aggarwal, D Vinoth Kanna, Akanksha A. Sharma, L. Badareesh, Vijayendra Kedage, Krishna Kalyan Reddy Jamunpalli, Sumit Arora, Gunjan Mishra, Yashwant Sakaray, Siddhant Khare, Bhakti Sarang Priti Patil, Prashant Bhandarkar

**Affiliations:** 1 WHO Collaborating Centre for Research in Surgical Care Delivery in LMICs, Mumbai, India; 2 Department of Surgery, Terna Medical College and Hospital, New Mumbai, India; 3 Gynaec-Oncology and Obstetrics, Command Hospital, Kolkata, India; 4 Bhabha Atomic Research Centre (BARC) Hospital, Mumbai, India; 5 Department of Surgery, Bhabha Atomic Research Centre (BARC) Hospital, Mumbai, India; 6 Department of Surgery, Maulana Azad Medical College, New Delhi, India; 7 Department of Surgery, Seth G.S. Medical College and KEM Hospital, Mumbai, India; 8 Department of Surgery, Manipal Hospital, Delhi, India; 9 Christian Medical College and Hospital, Ludhiana, India; 10 Baroda Medical College, Vadodara, India; 11 School of Health System Studies, Tata Institute of Social Sciences, Mumbai, India; 12 Department of Surgery, Post Graduate Institute of Medical Education and Research, Chandigarh, India; 13 Global Surg Research Collaborative, University of Birmingham, Birmingham, UK; 14 Department of Paediatric Surgery, Christian Medical College, Ludhiana, India; 15 Department of Obstetrics and Gynaecology, Christian Medical College, Ludhiana, India; 16 Department of Surgery, Government Medical College and Rajindra Hospital, Patiala, India; 17 Department of Surgery, Dr Hedgewar Arogya Sansthan, Delhi, India; 18 Department of Surgery, Kasturba Medical College and Hospital, Manipal, India; 19 Department of Surgery, Rao Tula Ram Hospital, Delhi, India; 20 Department of Surgery, Government Medical College, Trivandrum, India; 21 Northwestern University Feinberg School of Medicine, Illinois, USA; 22 Department of Global Public Health, Karolinska Institutet, 171 77 Stockholm, Sweden

## Abstract

**Background:**

Cancellations of elective surgeries on the day of surgery (DOS) can lead to added financial burden and wastage of resources for healthcare facilities; as well as social and emotional problems to patients. These cancellations act as barriers to delivering efficient surgical services. Optimal utilisation of the available resources is necessary for resource-constrained low-and-middle-income countries (LMIC). This study investigates the rate and causes of cancellations of elective surgeries on the DOS in various surgical departments across ten hospitals in India.

**Methods:**

A research consortium ‘IndSurg’ led by World Health Organisation Collaboration Centre (WHOCC) for Research in Surgical Care Delivery in LMICs, India conducted this multicentre retrospective cross-sectional study to analyse the cancellations of elective/planned surgical operations on DOS across urban secondary and tertiary level hospitals. We audited surgical records of a pre-decided period of six weeks for cancellations, documented relevant demographic information and reasons for cancellations.

**Results:**

We analysed records from the participating hospitals, with an overall cancellation rate of 9.7% (508/5231) on the DOS for elective surgical operations. Of these, 74% were avoidable cancellations. A majority (30%) of these 508 cancellations were attributed to insufficient resources, 28% due to patient's refusal or failure to show-up, and 22% due to change in patient's medical status.

**Conclusion:**

We saw a preponderance of avoidable reasons for elective surgery cancellations. A multidisciplinary approach with adequate preoperative patient counselling, timely communication between the patients and caregivers, adequate preoperative anaesthetic assessment, and planning by the surgical team may help reduce the cancellation rate.

## Introduction

Efficient delivery of surgical services is a key performance indicator of healthcare establishments. It requires significant resource allocation and high-level organisational efforts which is a reflection of the overall effectiveness and proficiency of the hospital management systems [1]. Elective surgery cancellations on the ‘day of surgery’ (DOS) may result from inadequate preoperative patient evaluation, inefficient patient counselling and organisational, administrative and operating room fallacies. Thus, these cancellations are an indirect indicator of patient care quality and a marker to assess the hospital management systems [1, 2]. The DOS cancellations lead to serious social, financial and administrative consequences on health care systems with an additional emotional burden on patients and their families [3]. The resulting economic losses incurred are detrimental to both the patient and the hospital. A United Kingdom study estimated an annual loss of 88 million dollars incurred by the National Health System owing to last-minute surgery cancellations [4]. A West African study from Burkina Faso estimated an additional cost of 19 thousand dollars incurred over three months by the hospital, with a mean value of 80 dollars per patient due to surgery cancellations [5]. Such a substantial economic impact on the healthcare systems necessitates efforts towards identifying the various factors related to such cancellations. Most low-and-middle-income countries (LMICs) exhibit a relatively higher rate (30–40%) of surgical cancellations as compared to the majority of the high-income countries (HICs). Although cancellation rates ranging between 1 and 24% have been reported from HICs, most are less than 10%, towards the lower end of the spectrum [6]. A high burden of surgical diseases in LMICs coupled with a lack of resources makes it crucial for LMICs to deliver more efficient surgical services to improve patient outcomes [7].

An integrated plan to identify and address the reasons for cancellations and ensure optimal utilisation of existing resources will help in delivering surgical care more effectively to the masses. It may contribute towards achieving 'Global surgery 2030' targets of provision of timely access to safe and affordable surgical care for LMICs like India [8, 9].

The diverse reasons for elective surgery cancellations range from inadequate preoperative assessment and preparation, patient-related factors, financial or administrative reasons, non-availability of personnel or equipment, lack of operating room time, and emergency surgery disrupting the elective list [6, 10, 11]. However, studies emphasising the rates of elective surgical cancellations and documenting the reasons from India are mainly single-centre studies [12, 13]. Therefore, we conducted this study to identify the current rate and reasons for surgical cancellations on the DOS across various health centres in India.

## Methodology

### Research collaboration

A research consortium ‘IndSurg' led by World Health Organisation Collaboration Centre (WHOCC) for Research in Surgical Care Delivery in LMICs, India, conducted this retrospective cross-sectional study in public and private hospitals across India. The participating hospitals provided elective and emergency surgical services in General Surgery, Orthopaedics, and Obstetrics-Gynaecology departments which offered a bulk of essential surgical services [14].

We collected the data of planned elective surgeries scheduled from 1st November 2019 to 15th December 2019. This time frame of 6 weeks was decided keeping in mind the resource constraints and non-availability of funds for data collection over a longer duration across the participating hospitals. At the same time, it provided a substantial number of surgeries for analysis. We analysed the data for the surgical operations performed and the ones cancelled on the scheduled DOS. Postponement of surgery or cancellations were both categorised as ‘cancelled’ for the planned DOS. We compiled the reasons for these surgery cancellations and then anonymised data as per predetermined categories. We decided the categories through literature review based on prospective or retrospective audits of elective surgical cancellations and discussed them between the team members for uniformity. These reasons were preoperative workup-related, patient compliance or follow-up related, change in the patient's medical status, financial or administrative, resource/equipment related, and workforce-related [6, 10, 11].

**Fig. 1:**
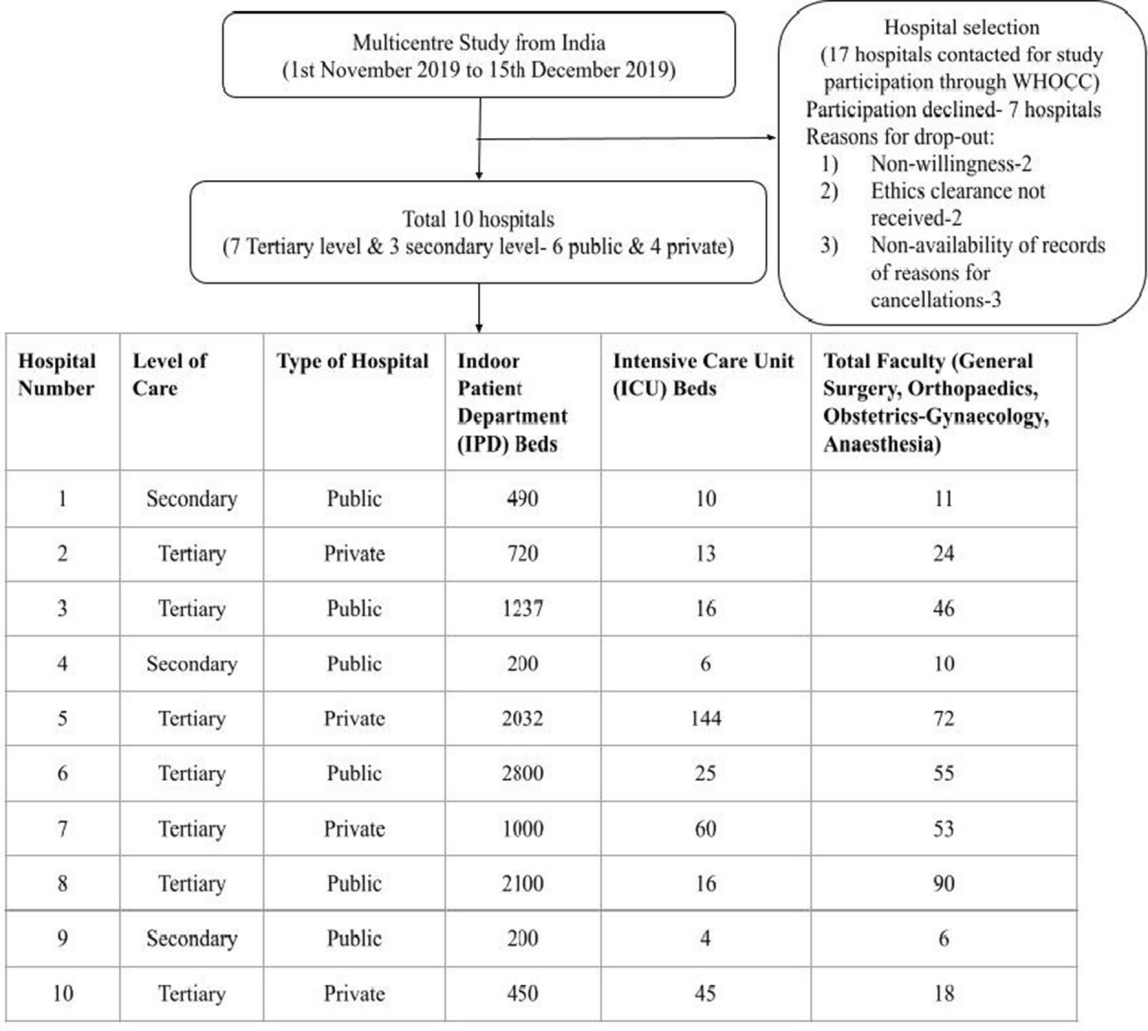
Recruitment algorithm and hospital characteristics

We sent invitation letters to the departmental heads of 17 hospitals from India. The hospitals were selected as per convenience sampling by the principal investigators. Of the 17 hospitals contacted, ten agreed to participate, of which seven were tertiary care hospitals and three were secondary care centres. Six of the ten hospitals were public hospitals while four were private hospitals. The names of these hospitals with the level of care provided and type (public or private) have been documented in Table 1. All the hospitals obtained Institutional Ethics Committee approval locally. The recruitment algorithm with the hospital characteristics is shown in Fig. 1.

**Table 1:** Names of hospitals with the level of care and type

Name of hospital	Level of care	Type of hospital
Christian Medical College and Hospital, Ludhiana	Tertiary	Private
Government Medical College and Rajindra Hospital, Patiala	Tertiary	Public
Kasturba Medical College and Hospital, Manipal	Tertiary	Private
Maulana Azad Medical College and Lok Nayak Hospital, New Delhi	Tertiary	Public
Mahatma Gandhi Mission Medical College and Hospital, New Mumbai	Tertiary	Private
Post Graduate Institute of Medical Education and Research, Chandigarh	Tertiary	Public
Terna Medical College and Hospital, New-Mumbai	Tertiary	Private
Bhabha Atomic Research Centre Hospital, Mumbai	Secondary	Public
Dr. Hedgewar Arogya Sansthan, Delhi	Secondary	Public
Rao Tularam Memorial Hospital, Delhi	Secondary	Public

### Data collection

The operation theatre (OT) scheduling at all these hospitals is on a first-come-first-serve basis considering the nature and urgency of the disease. Most patients scheduled for elective surgeries undergo preoperative investigations on an out-patient basis. An anaesthetist evaluates them to check if a patient is fit to undergo the desired surgical procedure before scheduling. An OT schedule is prepared at all these centres a day before the intended DOS. Based on the planned surgery, patients are admitted at least one day before the surgery. The investigator teams at these institutes collected data on the surgeries performed and the cancellations from the hospitals’ electronic medical records, OT booking log-books, or records from nurses' registers maintained in the OTs. The reasons for cancellations at most of the centres were documented by the surgeons and corroborated by the nursing staff.

The details of the hospitals included were name, level of care provided, number of beds (intensive care units and wards), number of available OTs, and the total number of faculty in the specialities of general surgery, obstetrics-gynaecology, orthopaedics, and anaesthesia (Fig. 1). Data collected were department names, age, sex, date of the surgery, details on preoperative workup, type of anaesthesia planned, whether the surgery was performed, cancelled, or postponed on the scheduled day, and the reasons cited for the cancellation or postponement.

### Data categorisation

We blinded the patient identification and coded the hospital names. The data analysis team collated and categorised the reasons for cancellations. We derived the rate of surgical cancellations with the total number of elective surgeries planned during the study period as the denominator. The cancellations were categorised as per six predetermined categories which were further stratified into ‘avoidable’ which were definitely correctable reasons and ‘potentially avoidable’ in which there may be a scope of improvement, but at times may not be prevented in spite of imparting the best level of care. Weekly telephonic meetings were held with collaborators and we resolved any discrepancies through mutual discussions till consensus was reached.

Our primary outcome measure was ‘the rate of cancellations of elective surgery’ on the DOS. The secondary outcome measures were: ‘the reasons for surgery cancellations’, to stratify the reasons based on whether those were ‘avoidable’ or ‘potentially avoidable’.

### Data analysis

We analysed data with Microsoft Excel 2019 and RStudio for Windows (Version 3.1.7, R Working Group). The qualitative variables were presented in frequencies followed by the cancellation percentage. Chi-square test was used to compare proportions and *p*-value of less than 0.05 was considered as statistically significant. Data were summarised in the form of frequencies of the reasons for cancellations.

## Results

A total of 5231 elective surgeries were planned. Of these, 508 surgeries were cancelled on the DOS, with an overall rate of surgery cancellations of 9.7%. Table 2 describes the demographic details of the patients in whom the surgeries were cancelled. Cancellation rates were similar in all age groups. More cancellations occurred in male patients (11.2%) and orthopaedic surgeries. Also, cancellations were statistically more for patients needing general anaesthesia (GA) exclusively or in combination with regional anaesthesia. The percentage of surgery cancellations was non-significantly higher in public hospitals (10.3%) as compared to private hospitals (9.2%).

**Table 2:** Surgical cancellations on the day of surgery

Variables	Completed	Cancelled	Total	Percentage cancellation (%)	*P*-value
	4723	508	5231	9.7	
Gender-based					
Female	2218	194	2412	8.0	< 0.001
Male	2502	314	2816	11.2	
Not available	3	0	3	0.0	
Age-group based					
0–14 years	267	24	291	8.2	0.16
15–29 years	964	95	1059	9.0	
30–44 years	1433	139	1572	8.8	
45–59 years	1124	126	1250	10.1	
60–74 years	646	83	729	11.4	
> 75 years	289	41	330	12.4	
Department-based					
General surgery	2494	249	2743	9.1	< 0.001
Obstetrics—gynaecology	658	33	691	4.8	
Orthopaedics	1571	226	1797	12.6	
Anaesthesia type-based					
General anaesthesia	1948	230	2178	10.6	< 0.001
Regional	2018	166	2184	7.6	
Local anaesthesia	703	29	732	4.0	
Combined	8	1	9	11.1	
Missing data	46	82	128		
Hospital setting					
Public	2148	246	2394	10.3	0.21
Private	2575	262	2837	9.2	

The reasons for surgery cancellations have been listed in Table 3. Around 30% of surgical cancellations were due to insufficient resources, 28% were due to patient compliance or follow-up-related factors, and 22% were due to change in patient's medical status. The most common reason for surgery cancellations in the public hospitals was resource/material relate (50.8%) whereas, in the private hospitals, surgeries were cancelled due to patient compliance-related factors (47.3%), details depicted in Table 3.

**Table 3:** Reasons for cancellation of surgery

Reason for cancellation	Public hospitals *n* (%)	Private hospitals *n* (%)	Overall *n* (%)
Resource/material-related	125 (50.8)	30 (11.5)	155 (30.5)
‘Over-run’ of OT time/lack of OT time	91 (37.0)	17 (6.5)	108 (21.3)
Emergency surgery prioritised	14 (5.7)	6 (2.3)	20 (3.9)
Equipment/Instrument unavailable/failure	13 (5.3)	6 (2.3)	19 (3.7)
Lack of bed in ICU (for Postoperative care)	7 (2.8)	1 (0.4)	8 (1.6)
Patient compliance-related	19 (7.7)	124 (47.3)	143 (28.1)
Patient failed to show-up	14 (5.7)	84 (32.1)	98 (19.3)
Patient/family refused surgery	5 (2.0)	40 (15.3)	45 (8.9)
Change in patient’s medical status	57 (23.2)	55 (21.0)	112 (22.0)
Manpower/human resources	67 (27.2)	4 (1.5)	71 (14.0)
Surgeon unavailability	25 (10.2)	4 (1.5)	29 (5.7)
Anaesthetist unavailability	28 (11.4)	0 (0)	28 (5.5)
Other staff-not available	14 (5.7)	0 (0)	14 (2.8)
Preoperative work-up-related	30 (12.2)	39 (14.9)	69 (13.6)
Incomplete evaluation/surgical work-up	22 (8.9)	31 (11.8)	53 (10.4)
Preoperative instructions not followed/NPO status	8 (3.3)	8 (3.1)	16 (3.1)
Financial/administrative	8 (3.3)	23 (8.8)	31 (6.0)
No financial/administration clearance	8 (3.3)	23 (8.8)	31 (6.0)
Other	5 (2.0)	10 (3.8)	15 (3.0)
Reason not mentioned	4 (1.6)	0 (0)	4 (0.8)

*OT* operation theatre, *NPO* nil per oral status

*Some surgeries had more than one reason for cancellation

Approximately 74% of cancellations of elective surgeries were avoidable and reasons for the same, stratified based on ‘avoidable’ and ‘potentially avoidable’, are depicted in Table 4.

**Table 4:** Day of surgery (DOS) cancellations stratified based on ‘Avoidable’ vs ‘Potentially avoidable’ causes

Categorical reasons of cancellations (*N* = 5231)	‘Avoidable’ causes of cancellations	‘Potentially avoidable’ causes of cancellations
Resource/material-related (30.5%)	Lack of OT time/time over-run (21.3%)	Emergency surgery prioritised (3.9%)
	Equipment/instrument unavailable/ failure (3.7%)	
	Lack of bed in ICU (for post op) (1.6%)	
Patient’s compliance-related (28.1%)	Patient failed to show-up (19.3%)	
	Patient/ family refused surgery (8.9%)	
Change in patient’s medical status (22.0%)		Change in medical status on DOS (22.0%)
Manpower/human resources (14.0%)	Surgeon unavailable (5.7%)	
	Anaesthetist unavailable (5.5%)	
	Other staff-not available (2.8%)	
Pre-operative work-up related (13.6%)	Incomplete evaluation/ surgical work-up (10.4%)	
	Preoperative instructions not followed/ NPO status (3.1%)	
Financial/administrative (6.0%)	No financial/administration clearance (6.0%)	

## Discussion

We found a surgery cancellation rate of 9.7% with 74% of these cancellations being avoidable. Literature globally has reported the incidence of surgical cancellations from under 1% to 40% [2, 3, 6, 11, 12]. A study from Spain reported a rate of 3.6%, while a study from the United States exhibited a cancellation rate of less than 2% [2, 15]. On the contrary, a study from the United Kingdom National Health Service hospitals has reported surgery cancellation rate as high as 13.9% [16]. However, a single-centre study from a public hospital in India reported an even higher rate of 17.6% of surgery cancellations [12]. Other LMIC studies from Malawi and Ethiopia have also reported surgical cancellation rates of around 30–40% [7, 17]. Although there are no definite standards for DOS cancellation rates, studies have reported centres with less than 5% cancellation rates as efficient [16, 18]. Owing to the robust healthcare infrastructure and management systems, most HICs are seen to perform better in terms of DOS cancellations' reported rates. Low rates of surgery cancellations can serve as a marker for improved quality of care, thus resulting in the efficient use of hospital resources with reduced patient care costs [19]. As Indian hospitals strive to achieve this, we must look into the factors that lead to relatively higher cancellation rates. Our multicentre study found an over-run of OT time (21.3%), patients failing to show-up for admission (19.3%), and a last-minute change in the patients' medical condition (22.0%) as the most frequent reasons. Similar findings were noted in other Indian studies, with 72% of cases cancelled due to lack of OT hours and reports of scheduling errors and overbooking in anticipation of unexpected cancellations [12, 20].

Optimal utilisation of OTs is a complex process, both clinically and operationally. At times, the over-run of the OT schedule may be due to unprecedented events or complications during the surgery. Yet some of the processes in this complex OT exercise are amenable to improvements [1]. Underestimation of surgery time by the surgeons, prolonged waiting time between cases due to delay in OT preparation, patient transportation and delay in initiation of the first case without compensating for the lost time in the latter part of the day are vital areas where the multidisciplinary team can improve on. Fixed duty hours, lack of monetary incentives for working beyond the scheduled duty hours, and insufficient staffing may be other factors responsible. Regular OT team meetings to debrief on cancellations involving all the multidisciplinary stakeholders and time stamping of each of the OT processes may help improve time utilisation [21].

Patients failing to show-up on the DOS was the second most common reason for cancellations. It may be due to lack of patient education, fear or unwillingness for the surgery, patients forgetting the date of surgery, seeking treatment at another hospital due to perception of or actual delay in surgical scheduling, miscommunication by the treating surgeons, or preference for hospitals providing the surgical service at a relatively low cost. Comprehensive patient counselling by the surgical team or designated trained liaison person before surgery can mitigate these concerns [6]. Also, a security deposit may be a deterrent that can be used in most hospitals.

Change in a patient's medical status on the DOS may be caused by inadequate preoperative evaluation, however, they may also arise from an unexpected change in the patient's comorbidities [6, 12]. Although studies have documented that data regarding the quality of preoperative assessment and the waiting period between the assessment and DOS may influence the patient's medical state [22], a close liaison with the treating physician, surgeons, anaesthetists, and nursing personnel may help alleviate it [12]. A preoperative re-evaluation by the anaesthetists a day prior may help curtail certain potentially avoidable medical alterations.

Our study demonstrated more cancellations in surgeries planned under GA (10.6%). Studies have documented that a structured preoperative assessment and patient optimisation led to fewer surgery cancellations in cases posted under GA [12, 23]. Patient induction under GA is more resource-intensive and necessitates optimal patient preparation as compared to regional anaesthesia. The anaesthetists also prefer to avoid inducing GA cases during the latter part of the day. These cases usually run for a longer duration and require extended postoperative recovery observation. Less staffing in the later part of the day also acts as a deterrent. Adequate preoperative preparation and addressing the manpower constraints with an allocation of duties in shifts may help resolve this concern.

On the evaluation of surgery cancellation rates between the public and private hospitals, no significant difference was observed in the overall cancellation rates. However, on exploring the reasons for cancellations, ‘over-run of OT time’ was the most common reason in public hospitals whereas ‘patients failing to show up on the intended DOS’ was commonly seen in the private hospitals. Public hospitals in India are meant to reach out healthcare to vast sections of the underserved population and are essentially free [24, 25]. The overwhelming burden of elective surgical workload coupled with a dearth of infrastructure, overscheduling of cases, underestimation of surgical time, shortage of supporting staff in the OTs, slow turnover of cases due to most hospitals being teaching institutes and time lost between cases due to slow processes may be the factors responsible for the surgery cancellations in these hospitals [13, 24]. Private hospitals in India are also responsible for the provision of a large part of healthcare and the healthcare expenses are paid out of pocket by the patients [26]. Patients failing to show-up may be explained by the cost of care. But other factors like lack of appropriate communication about the surgical dates, fear about the surgery and a scheduling delay cannot be ruled out.

### Limitations

The selection of hospitals followed a convenient sampling method which may have introduced a selection bias. Due to the short 6-weeks period, the seasonal variations in the surgical workload may have been missed. This is a unique situation in India, wherein patients have a misconception of better wound healing during the winter months than the hot summer weather [27]. This leads to increased surgical volumes during the winter months (study period) which may overwhelm the existing surgical services leading to increased cancellations. Also, although this study has succeeded in providing glimpses of the reasons responsible for surgery cancellations, further in-depth analysis is warranted with prospective, longer-duration studies.

## Conclusions

This study demonstrated a surgical cancellation rate of 9.7%, which is higher than the majority of the healthcare systems in HICs and the reported acceptable standards of efficiency. We observed that most of the surgery cancellation determinants were avoidable. A multidisciplinary approach with adequate preoperative patient counselling, optimal assessment by anaesthetists in close liaison with surgeons, and rigorous preoperative surgical planning may help improve the utilisation of hospital resources and reduce cancellations. However, further in-depth analysis with prospective, longer-duration studies may help in identifying definitive solutions.
